# Machine learning based insights into cardiomyopathy and heart failure research: a bibliometric analysis from 2005 to 2024

**DOI:** 10.3389/fmed.2025.1602077

**Published:** 2025-07-25

**Authors:** Muhammad Junaid Akram, Asad Nawaz, Yuan Yuxing, Jinpeng Zhang, Huang Haixin, Lingjuan Liu, Xu Qian, Jie Tian

**Affiliations:** ^1^Ministry of Education Key Laboratory of Child Development and Disorders, Department of Pediatric Cardiology, National Clinical Key Cardiovascular Specialty, National Clinical Research Center for Child Health and Disorders, Children’s Hospital of Chongqing Medical University, Chongqing, China; ^2^Key Laboratory of Children’s Important Organ Development and Diseases, Children’s Hospital of Chongqing Medical University, Chongqing Municipal Health Commission, Chongqing, China; ^3^Library, Chongqing Medical University, Chongqing, China

**Keywords:** cardiomyopathy, heart failure, bibliometric, VOSviewer, CiteSpace, machine learning, artificial intelligence

## Abstract

**Background:**

Cardiomyopathy and heart failure are among the most critical challenges in modern cardiology, with increasing attention to the integration of machine learning (ML) and artificial intelligence (AI) for diagnostics, risk prediction, and therapeutic strategies. This study was aimed at evaluating global research trends, influential contributions, and emerging themes in the domain of cardiomyopathy and heart failure from 2005 to 2024.

**Methodology:**

A comprehensive bibliometric analysis was conducted using the Web of Science Core Collection (WoSCC) database. The study utilized the R- package bibliometrix-biblioshiny, VOSviewer, Scimago Graphica and CiteSpace to analyze the publications on cardiomyopathy, heart failure, machine learning, and artificial intelligence. Key metrics examined included top institutions, countries, journals, keywords, co-authorship networks, and keyword co-occurrence patterns. Additionally, the analysis evaluated publication counts, citation trends, H-index, and collaboration metrics to identify research trends and emerging themes in the field.

**Results:**

A total of 2,110 publications retrieved from the last 20 years were included in the analysis. The United States of America (USA), China, and the United Kingdom (UK), emerged as leading contributors, with institutions such as Mayo Clinic and Harvard University producing high-impact research. Dominant keywords included “heart failure,” “risk,” “diagnosis,” and “artificial intelligence,” reflecting the increasing reliance on ML for predictive analytics. Thematic evolution revealed a transition from traditional classification methods to advanced techniques, including feature selection and proteomics. Influential studies, including those by Friedman PA, Noseworthy PA, and Attia ZI, showcased the transformative potential of AI in cardiology. Global collaboration networks underscored strong partnerships but highlighted disparities in contributions from low-income regions.

**Conclusion:**

This analysis highlights the dynamic evolution of cardiomyopathy research, emphasizing the critical role of ML and AI in advancing diagnostics and therapeutic strategies. Future research should address challenges in scalability, data standardization, and ethical considerations to ensure equitable access and implementation of these technologies, particularly in underrepresented regions.

## Introduction

Cardiovascular disease (CVD) is the primary contributor to global morbidity and mortality, placing a substantial burden on affected individuals and society as a whole (1–3). Cardiomyopathy (CM) is a relatively rare and often-overlooked yet severe form of CVD, characterized by cardiac structural abnormalities, arrhythmias, and potential progression to heart failure (4). CM can lead to the abrupt death of individuals affected during childhood or adolescence, and in certain instances, patients may need to undergo cardiac transplantation (5). In 2019, the global age-standardized prevalence rate of CM was 56.0 per 100,000 persons, with 0.71 million cases of alcoholic and 3.73 million cases of other cardiomyopathies (6), whereas population-based studies in the United States, Australia, and Finland estimated an incidence of 1.00 per 100,000 person-years in children (7–9).

The global burden of CM and myocarditis in older adults aged 60-89 was significant, with 475,458 incidence cases and 185,308 deaths in 2019 (10). Cardiomyopathy is a major contributor to the global burden of heart failure, which affects over 37.7 million individuals worldwide; in 2019, cardiomyopathy-related heart failure accounted for 3.37 million disability-adjusted life years (DALYs) and an age-standardized incidence rate of 16 per 100,000 person-years among older adults aged 60–89 years (10–13). Despite the significant global burden of cardiomyopathy and heart failure, early research predominantly utilized traditional analytical models, limiting progress in advancing diagnostic, prognostic, and therapeutic strategies. The advent of machine learning (ML) and artificial intelligence (AI) has led to substantial improvements in risk stratification, diagnostic precision, and predictive modeling by enabling the analysis of complex datasets and uncovering intricate disease mechanisms beyond the scope of conventional methods (14–18). This bibliometric analysis was designed to analyze global publication trends, leading contributors, evolving thematic priorities, and collaborative patterns in machine learning and artificial intelligence research related to cardiomyopathy and heart failure between 2005 and 2024. The novelty of this work lies in its dual focus on cardiomyopathy and heart failure, its application of three advanced bibliometric tools (VOSviewer, CiteSpace, and Bibliometrix), and its extended temporal coverage. In contrast to prior reviews (19–21), this study offers a broader perspective by incorporating contextual dimensions including ethical challenges, technological evolution, and the underrepresentation of low- and middle-income regions in this research landscape.

## Methodology

### Data collection

**FIGURE 1:**
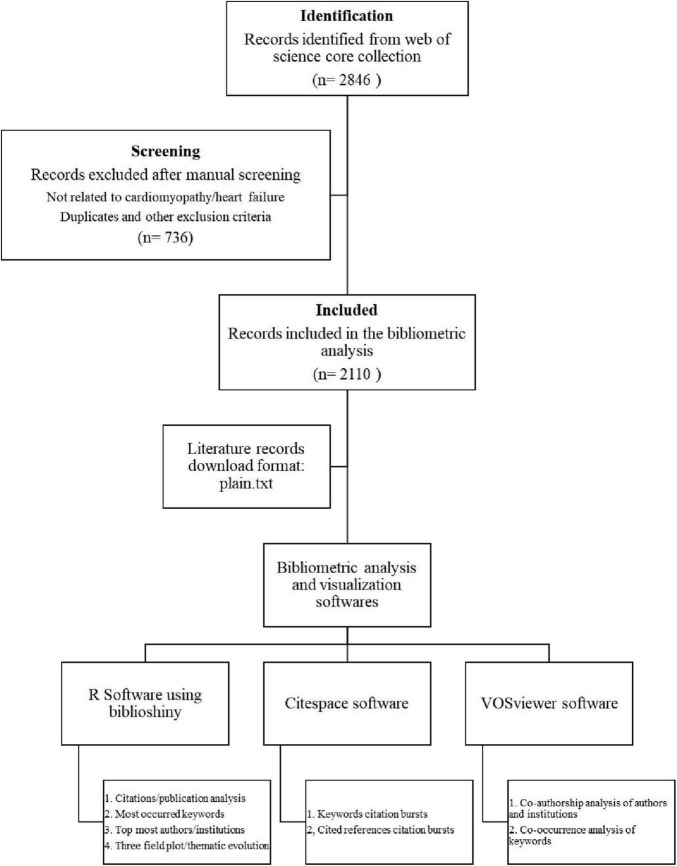
Bibliometric flow chart.

The Science Citation Index Expanded (SCIE) within the Web of Science Core Collection (WoSCC) (22) was utilized as the target database to retrieve research articles published between January 2005 and December 2024 (Figure 1). To ensure precision and avoid irrelevant results, we employed a highly specific search strategy targeting the most commonly used terms in the literature: TS = (“Machine Learning” OR “Artificial Intelligence”) AND TS = (“Cardiomyopathy” OR “Heart Failure”). This approach was chosen to align with the narrow scope of our research question and to retrieve studies directly relevant to our topic. Only articles and review articles published in English language were included in the analysis, while publications with titles in non-English languages were excluded. Additionally, conference abstracts, news articles, editorials, meeting reports, and case reports were excluded from consideration. To minimize selection bias, all eligible articles were downloaded in a single session. Two authors independently performed manual screening of titles and abstracts to exclude irrelevant studies (i.e., those unrelated to cardiomyopathy or heart failure) and duplicates. The process yielded a final dataset of 2,110 records for bibliometric analysis. The references were exported in plain text format and stored as download.txt files for subsequent analysis (23).

### Data analysis

We imported all the downloaded data into VOSviewer (version 1.6.20), Scimago Graphica, R-studio using Biblioshiny (version 4.4.1), and CiteSpace (version 6.4.R1) software for running the bibliometric analysis. VOSviewer is a widely used software for constructing, visualizing, and analyzing bibliometric and network-based data, making it particularly valuable in medical and scientific research. It facilitates the creation of networks from bibliographic databases and provides advanced visualizations, including network, overlay, and density maps, for the detailed exploration of complex datasets. In this study, co-author analysis was employed to reveal collaborative relationships among authors and institutions, while co-occurrence analysis highlighted associations between different keywords. Additionally, the analysis was enhanced by visualizing the network over time using VOSviewer’s time overlay feature (24–26).

CiteSpace is a bibliometric visualization tool widely utilized in scientific research to map knowledge domains, analyze citation patterns, and identify emerging trends. It employs advanced techniques such as cluster analysis, timeline views, and citation burst detection to uncover widely cited references and keywords within a specified timeframe (27). In this study, CiteSpace was used to identify highly cited references and keywords with strong citation bursts within a specific time frame. The software provides valuable insights into emerging trends across various disciplines (28).

Additionally, we also used RStudio, developed by Posit Software, which serves as an integrated development environment (IDE) for R, enabling efficient statistical computing and data visualization. The biblioshiny application, included in the bibliometrix R package, provides a user-friendly, web-based platform for conducting advanced bibliometric analyses. It supports the integration of bibliographic data from major databases, facilitating citation analysis, co-authorship network mapping, and keyword co-occurrence studies (29–31).

## Results

### The trends of citations and publication analysis

**FIGURE 2:**
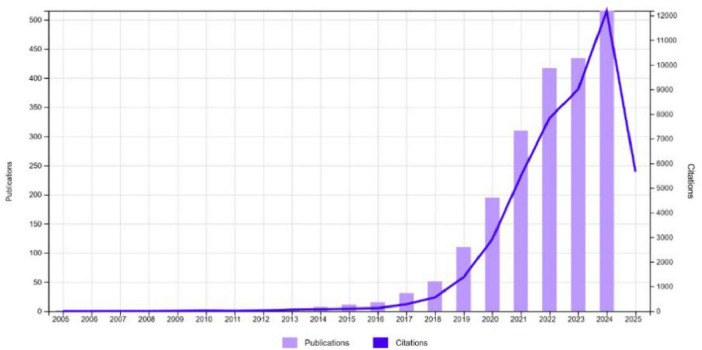
Annual number of publications and citations.

Figure 2 demonstrates a significant growth in publications and citations in cardiomyopathy and heart failure research from 2005 to 2024. While research output remained minimal until 2015, a rapid increase in publications began in 2016, exceeding 500 by 2024, showing a growth rate of 20.25% annually. Citations also rose sharply, surpassing 12,000 by 2024, reflecting the growing impact and recognition of research in this field. This post-2016 publication surge aligns with broader developments in AI research, including the introduction of deep learning frameworks, increasing availability of big data in healthcare, and landmark cardiology studies demonstrating ML’s clinical utility. It also reflects a rise in interdisciplinary collaborations and greater institutional and funding support for digital health innovations. This trend highlights the increasing scientific interest and the critical importance of advancing knowledge in machine learning based cardiomyopathy and heart failure research.

### Countries-wise distribution analysis

**FIGURE 3:**
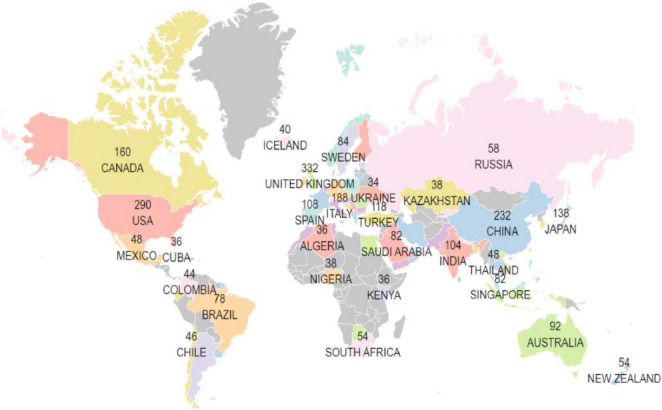
Country-wise affiliation count of publications.

The data illustrated that 85 countries and regions have published research in the field of machine learning and cardiomyopathy. The geographic map (Figure 3) shows the country or regions -wise affiliations count of institutes conducting research in this domain.

Additionally, Table 1A illustrates the distribution of corresponding authors’ countries, highlighting the number of single-country publications (SCP) and multiple-country publications (MCP) to evaluate international collaboration in cardiomyopathy and heart failure research. The United States of America (USA) leads with 631 publications, including 453 SCPs and 178 MCPs, reflecting an international collaboration rate of 28.2%.

**TABLE 1A:** Top 10 corresponding author’s countries and collaborations.

Rank	Country	Articles	Articles (%)	SCP	MCP	MCP (%)
1	USA	631	30	453	178	28.2
2	China	444	21	360	84	18.9
3	UK	127	6	46	81	63.8
4	Italy	89	4.2	55	34	38.2
5	Japan	74	3.5	62	12	16.2
6	Canada	59	2.8	35	24	40.7
7	Germany	55	2.6	19	36	65.5
8	India	53	2.5	37	16	30.2
9	Korea	49	2.3	37	12	24.5
10	France	41	1.9	18	23	56.1

SCP, Single country publication; MCP, Multi country publication.

China follows with 444 publications, with 360 SCPs and 84 MCPs, showcasing an 18.9% collaboration rate. The United Kingdom (UK) demonstrates the highest international collaboration rate at 63.8%, with 81 MCPs out of 127 total publications. Moreover, the annual publications and citations volume of the top ten countries during the last two decades is described in Table 1B. The USA and China account for more than 50% of the research publications and the relevant citations.

**TABLE 1B:** Top 10 countries contributing to publications and citations.

Rank	Country	Publications	Country	Citation	Average article citations
1	USA	3504	USA	23177	36.7
2	China	1833	China	4371	9.8
3	UK	830	UK	3399	26.8
4	Italy	586	Australia	1808	50.2
5	Germany	554	Italy	1623	18.2
6	Japan	416	Canada	1271	21.5
7	Canada	360	Korea	979	20
8	Spain	337	Spain	775	20.4
9	France	250	Germany	700	12.7
10	Australia	216	Japan	668	9

### Authors wise distribution analysis

The top ten most prolific authors (Table 2) in the field demonstrate significant contributions, with Friedman PA and Noseworthy PA leading in both publications (NP = 27 and 24, respectively) and citation metrics (TC > 2,500, H-index = 14). The analysis of bibliometric indices including the H-index, G-index, and M-index, provided additional insights into the productivity and impact of authors and journals in this field. The H-index measures the productivity and citation impact of an author or journal, where an index of H indicates that H papers have been cited at least H times each (32, 33). The G-index complements the H-index by giving more weight to highly cited papers, thus better reflecting the overall citation performance of prolific authors (34). The M-index is a time-normalized metric, calculated as the H-index divided by the number of years since the first publication, offering insights into the consistency and temporal trends of research impact (35). Authors including Attia ZI and Lopez-Jimenez F also exhibit high impact, with substantial citations (TC = 2,739 and 2,377, respectively) and notable H-index scores. Emerging contributors, including Li Y and Petersen SE, show promising research activity with consistent publication rates and growing indices since 2018.

**TABLE 2:** Top 10 authors and their local impact.

Rank	Author	NP	TC	H-index	G-index	M-index	PY
1	Friedman PA	27	2995	14	27	2	2019
2	Noseworthy PA	24	2895	15	24	2.14	2019
3	Attia ZI	21	2739	12	21	1.71	2019
4	Li Y	19	2346	10	20	1.667	2019
5	Lopez-Jimenez	19	2377	12	19	1.71	2019
6	Sengupta PP	18	1158	13	18	1.3	2016
7	Wang Y	16	250	7	15	0.583	2013
8	Zhang J	16	718	7	16	0.875	2017
9	Lin C	15	190	8	13	1.6	2021
10	Petersen SE	15	972	9	15	1.13	2018

NP, Number of publications; TC, Total citations; PY, Publication year.

**FIGURE 4:**
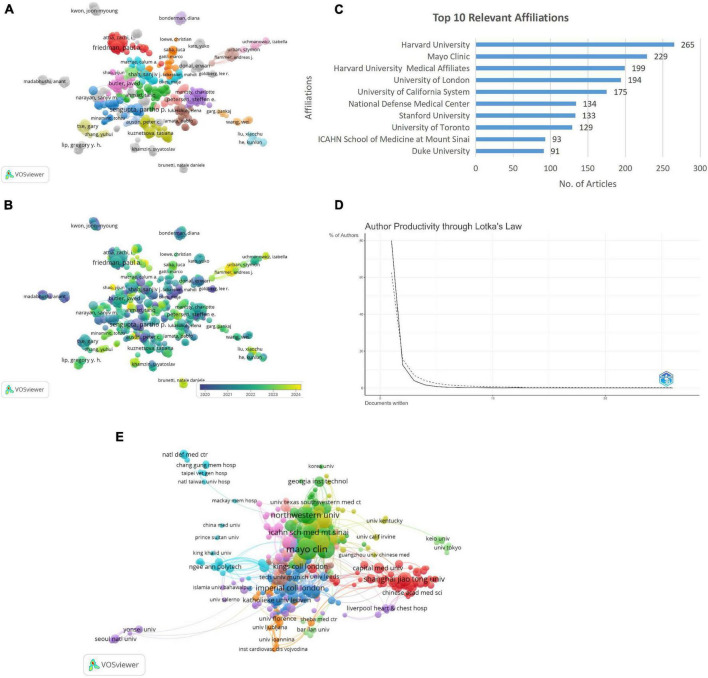
**(A)** Author-co-author network visualization. **(B)** Author co-author overlay visualization. **(C)** Top 10 relevant affiliation. **(D)** Author’s productivity through Lotka’s Law. **(E)** Institutional co-authorship analysis.

The author co-authorship network (Figure 4A) reveals key collaborative clusters (*n* = 35), with prominent authors including Friedman P.A. and Butler Javed forming central hubs. Whereas author co-authorship overlay visualization (Figure 4B) highlights the temporal dynamics of collaborations, with nodes in yellow representing recent contributions (2024) and blue indicating earlier activity (2020). Key authors Friedman P.A. and Butler Javed demonstrate sustained and evolving influence in the research network over time. The dominance of authors such as Friedman PA, Noseworthy PA, and Attia ZI, particularly from Mayo Clinic, reflects their pioneering work in AI-powered ECG (electrocardiogram) diagnostics and risk prediction. Their highly cited publications have served as reference points for subsequent studies and contributed to standardizing AI applications in cardiovascular care.

### Institutions wise distribution analysis

The analysis of institutional contributions highlights Harvard University as the leading affiliation with 265 articles, followed by Mayo Clinic, Harvard University medical affiliates, and the University of London with 229, 199, and 194 articles, respectively (Figure 4C). Application of Lotka’s Law revealed that 80% of authors contributed a single publication, while only 12.5% produced two publications, and less than 1% authored more than five publications. This finding aligns with Lotka’s principle of diminishing productivity, highlighting a concentration of research output among a small number of highly prolific authors (Figure 4D).

The Institutional Co-Authorship Analysis illustrates collaborative networks among research institutions, with nodes representing institutions and node sizes corresponding to their publication output. Links between nodes signify co-authorship relationships, where thicker links indicate stronger collaborations. Clusters are color-coded, grouping institutions with higher inter-collaboration. Notably, prominent institutions such as Mayo Clinic, Northwestern University, and Shanghai Jiao Tong University, Chinese academy of medical science, and Capital Medical University emerged as key contributors, reflecting their pivotal roles in fostering institutional collaborations in the field (Figure 4E). Mayo Clinic’s centrality in the collaboration network corresponds to its leadership in translating AI models into clinical workflows. Similarly, institutions like Harvard University and Northwestern University have been instrumental in driving methodological innovation and integrating ML into multi-center cardiology trials.

### Core sources and their impact analysis

The core journals identified through Bradford’s Law (Table 3A) represent Zone 1, encompassing the most productive sources with a total of 433 publications. Key contributors include Frontiers in Cardiovascular Medicine, Scientific Reports, and ESC Heart Failure with 111, 60, and 43 publications, respectively. The top 10 journals with the highest local impact in machine learning based cardiomyopathy and heart failure research (Table 3B) demonstrate Frontiers in Cardiovascular Medicine as the leading source, with 111 publications, an H-index of 16, and 951 total citations, followed by Scientific Reports and ESC Heart Failure BMC Medical Informatics and Decision Making stands out with the highest G-index of 28 and total citations of 1,478, reflecting its significant role in cardiomyopathy and heart failure research.

**TABLE 3A:** Core sources according to Bradford’s Law.

Journals	Rank	NP	Cum NP	Zone
Frontiers in cardiovascular medicine	1	111	111	Zone 1
Scientific reports	2	60	171	Zone 1
ESC heart failure	3	43	214	Zone 1
Journal of clinical medicine	4	42	256	Zone 1
Diagnostics	5	35	291	Zone 1
PLOS one	6	32	323	Zone 1
IEEE access	7	30	353	Zone 1
BMC medical informatics and decision making	8	28	381	Zone 1
International journal of cardiology	9	26	407	Zone 1
Journal of the american heart association	10	26	433	Zone 1

NP, Number of publications; Cum NP, Cumulative number of publications.

**TABLE 3B:** Top 10 sources and their local impact.

Source	H-index	G-index	M-index	TC	NP	PY
Frontiers in cardiovascular medicine	16	23	2.7	951	111	2020
Scientific reports	15	26	1.7	794	60	2017
ESC heart failure	10	21	1.4	484	43	2019
Journal of clinical medicine	13	18	1.6	413	42	2018
Diagnostics	10	14	2	263	35	2021
PLOS one	12	27	1	765	32	2014
IEEE access	11	28	1.48	829	30	2018
BMC medical informatics and decision making	11	28	1	1478	28	2015
International journal of cardiology	13	17	1.6	312	26	2018
Journal of the american heart association	13	26	1.6	721	26	2018

NP, Number of publications; TC, Total citations; PY, Publication year.

### Keywords occurrence and co-occurrence analysis

**FIGURE 5:**
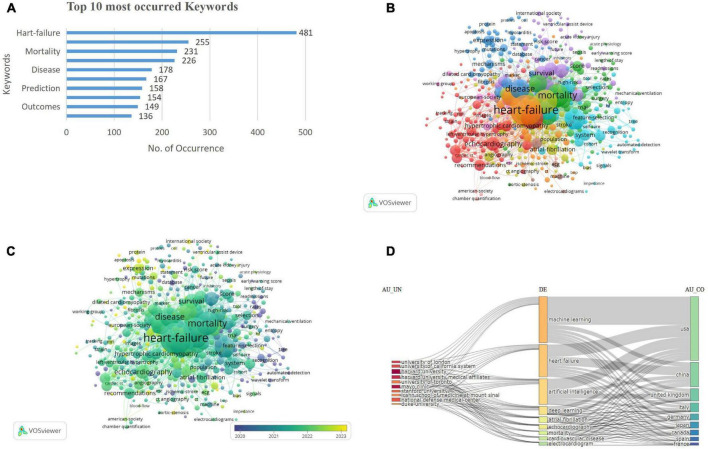
**(A)** Top occurred keywords. **(B)** Keywords co-occurrence network visualization. **(C)** Keywords co-occurrence overlay visualization. **(D)** Three-field plot of keyword analysis. [Left field: institutions (AU-UN); Middle field: keywords (DE); Right field: countries (AU-CO)].

The bibliometric analysis identified 3,648 key terms, with a predominant focus on “heart failure” (*n* = 481), followed by “risk” (*n* = 255), mortality (*n* = 231), and “diagnosis” (*n* = 266). Terms including “classification” (*n* = 167) and “prediction” (*n* = 158) emphasize the growing interest in predictive models and stratification, while “management” (*n* = 136) and “outcomes” (*n* = 149) reflect the focus on optimizing clinical care and improving patient prognosis (Figure 5A).

The keyword co-occurrence network visualization depicts 459 terms with at least five occurrences, organized into 10 clusters. Cluster 1 (red) included 83 keywords, encompassing terms such as echocardiogram, coronary syndrome, mortality, American society, and angiography. Cluster 2 (green), with 71 keywords, highlighted 30-day readmission, acute coronary syndrome, acute kidney injury, outcomes, and adults. Cluster 3 (dark blue) contained 62 keywords, including anticoagulation, cardiac hypertrophy, biomarkers, and cardiomyopathy. Cluster 4 (yellow) consisted of 54 keywords, including atrial fibrillation, aortic stenosis, preserved ejection fraction, and COVID-19. Cluster 5 (purple), with 51 keywords, emphasized models, logistic regression, risk scores, hospitalization, and precision medicine. Cluster 6 (light blue) comprised 50 keywords, featuring classification, systems, disease, prediction, and feature selection. Cluster 7 (orange), Cluster 8 (brown), Cluster 9 (violet), and Cluster 10 (pink) contained 46, 19, 16, and 7 keywords, respectively, covering diverse research themes, including clinical aspects (echocardiography, hypertrophic cardiomyopathy) and computational advancements (machine learning, feature selection), reflecting the interdisciplinary nature of cardiomyopathy research (Figure 5B). The prevalence of keywords such as “diagnosis,” “prediction,” and “risk” underscores the central aim of improving clinical decision-making through AI/ML. The emergence of terms like “classification,” “feature selection,” and “deep learning” highlights a methodological evolution from traditional analytics to sophisticated modeling techniques.

The overlay visualization of the keywords is shown in Figure 5C. The three-field chart visualizes the relationships between institutions (AU_UN), keywords (DE), and contributing countries (AU_CO). Prominent institutions like Harvard University and Mayo Clinic are linked to key topics including machine learning and heart failure, which are primarily driven by contributions from USA, China, and the UK. The chart highlights the collaborative and interdisciplinary nature of cardiology research across institutions, topics, and regions (Figure 5D).

### Research hotspots analysis

#### Most cited documents

The top-cited documents (Table 4) in the bibliometric analysis highlight significant contributions to cardiovascular research and medical informatics. Leading studies include Deo et al. (2015, Circulation, TC = 1,826, TC/Year = 182.6), Kornej et al. (2020, Circ Res, TC = 716, TC/Year = 143.2), and Attia et al. (2019, Lancet, TC = 759, TC/Year = 126.5). The data illustrates a general trend where the citation rate (TC/Year) is directly proportional to the total citations (TC), with some notable outliers. For instance, Siontis Kc (2021) in Nature Review Cardiology demonstrates a higher annual citation rate (82.4 TC/Year) compared to Bai Wj (2018) in the Journal of the American Medical Informatics Association (58.48 TC/Year), despite the latter being published earlier. This suggests that Siontis Kc’s work has had a more substantial impact in a shorter time span, underscoring the importance of considering TC/Year alongside TC to comprehensively assess research influence and identify significant contributions to the field.

**TABLE 4:** Most cited documents.

Documents	DOI	TC	TC/year
Doe RC, 2015, circulation	10.1161/CIRCULATIONAHA.115.00159	1826	182.60
Kornej J, 2020, Circ Res	10.1161/CIRCRESAHA.120.31634	716	143.20
Attia Zi, 2019, Lancet	10.1016/S0140-6736(19)31721-	759	126.50
Uddin S, 2019, BMC Med Inform Decis	10.1186/s12911-019-1004-	666	111.00
Attia Zi, 2019, Nat Med	10.1038/s41591-018-0240-	630	105.00
Johnson KW, 2018, J AM Coll Cardiol	10.1016/j.jacc.2018.03.52	569	81.29
Zhang J, 2018, Circulation	10.1161/CIRCULATIONAHA.118.03433	498	71.14
Krittana Wong C, 2017, J AM Coll Cardiol	10.1016/j.jacc.2017.03.57	517	64.63
Bai Wj, 2018, J Cardiovasc Magn R	10.1186/s12968-018-0471-	467	58.48
Siontis Kc, 2021, Nat Rev Cardiol	10.1038/s41569-020-00503-	412	82.4

TC, Total citations; TC/Year, Total citations per year; DOI, Digital object identifier.

### Keyword and reference bursts analysis

**FIGURE 6:**
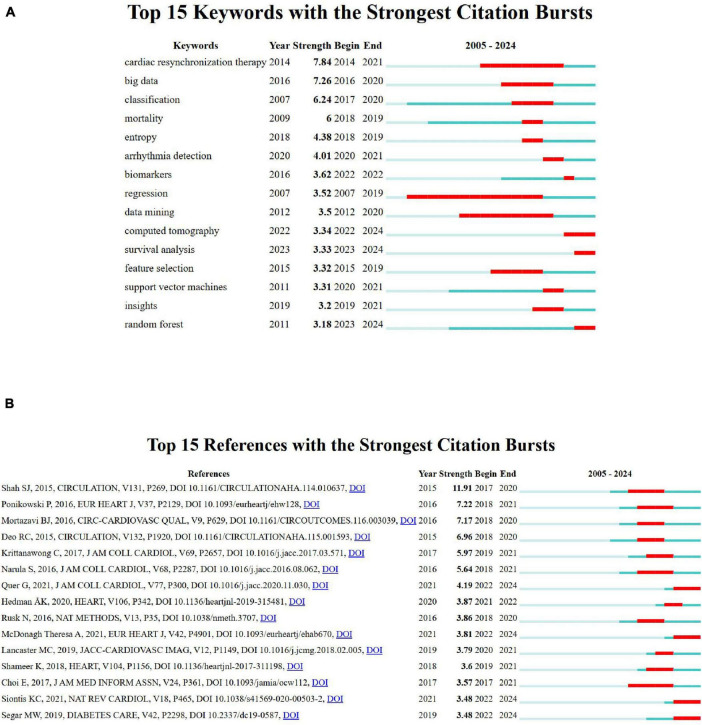
**(A)** Top 15 keywords with the strongest citation bursts. **(B)** Top 15 references with the strongest citation bursts.

The visualization (Figure 6A) highlights the top 15 keywords with the strongest citation bursts over the period 2005–2024. Red bars represent the timeframes during which each keyword experienced a significant surge in citations, known as a “citation burst,” indicating periods of heightened research interest. In contrast, blue bars denote intervals of stable or lower citation activity. The minimum burst range observed is 1 year, as seen for keywords like “mortality” and “entropy” (2018–20219). Keywords like “cardiac resynchronization therapy” with the highest burst strength (7.84), demonstrate exceptional attention between 2014 and 2021, while “regression”shows a prolonged burst (2007–2019), reflecting sustained interest. Emerging areas, such as “computed tomography” (2022–2024), signal newer research trends, while long-standing keywords like “data mining” (2012–2020) illustrate persistent relevance. These bursts reflect not only changing research priorities but also rapid adoption of AI tools in clinical cardiology. For example, the strong burst for “cardiac resynchronization therapy” aligns with studies exploring ML’s role in optimizing therapy timing and patient selection. Recent bursts in “computed tomography” suggest a growing integration of imaging and AI for non-invasive diagnostics.

Figure 6B highlights the top 15 references with the strongest citation bursts between 2005 and 2024, with significant influence seen in Shah SJ, 2015, Circulation (strength 11.91) and Ponikowski P, 2016, Eur Heart J (strength 7.2). The burst durations, represented by red bars, indicate peak research relevance, particularly for studies on statistical learning, cardiovascular imaging, and advanced cardiac care. Emerging trends are evident in the consistent citation bursts for computational and cardiovascular-related research.

### Funding agencies: public vs. private contributions

Analysis of funding revealed that public sector support predominated, accounting for 89% of the total funding. The United States Department of Health and Human Services and the National Institutes of Health were the leading contributors, followed by significant support from China’s National Natural Science Foundation and various European funding agencies. In contrast, private sector involvement represented 11% of funding, with pharmaceutical companies including AstraZeneca, Pfizer, and Novartis providing the majority of industry contributions, alongside funding from medical device manufacturers like Medtronic and Abbott Laboratories. The observed 8:1 ratio of public to private funding suggests that public sector investments predominantly support basic science and clinical research, while private sector funding is more focused on the development of therapeutic interventions. (Table 5) summarizes contributions from major agencies.

**TABLE 5:** Funding agencies.

Public funding agencies	Papers	Private funding agencies	Papers
United States department of health human services	302	AstraZeneca	27
National institutes of health NIH USA	290	Pfizer	20
National natural science foundation of China NSFC	164	Novartis	14
NIH National heart lung blood institute NHLBI	131	Medtronic	13
American heart association	68	Johnson & Johnson	18
UK research innovation UKRI	60	Bristol myers squibb	13
European union EU	56	Amgen	12
British heart foundation	52	Boehringer ingelheim	11
Medical research council UK MRC	37	Abbott laboratories	9
Ministry of education culture sports science and technology Japan MEXT	37	Bayer AG	8

### Thematic evolution analysis

**FIGURE 7:**
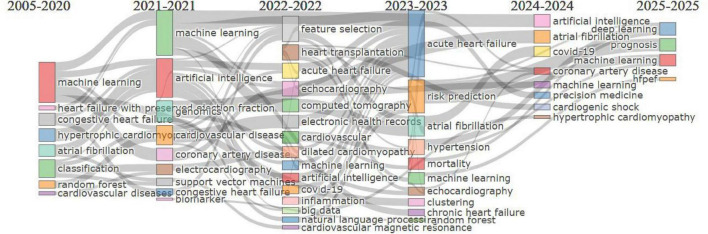
Thematic evolution map.

The thematic evolution map (Figure 7) illustrates the progression of research themes in cardiovascular and biomedical studies from 2005 to 2025. Early focus (2005–2020) was on foundational computational methods like “machine learning,” “classification,” and “congestive heart failure.” From 2021 to 2022, themes evolved to advanced technologies such as “artificial intelligence” and “feature selection” with clinical applications like “genomics” and “cardiovascular disease.” In 2023, diagnostic and predictive methods, including “echocardiography,” “random forest,” and “risk prediction,” gained prominence. By 2024, emerging areas like “immune cell infiltration,” “proteomics,” and “chronic disease” highlighted precision medicine. Finally, 2025 research integrates cutting-edge tools like “deep learning” and focuses on “telemedicine,” “prognosis,” and “bioinformatics,” reflecting the advancement toward data-driven, personalized care.

## Discussion

This bibliometric analysis provides a comprehensive evaluation of cardiomyopathy and heart failure research trends from 2005 to 2024, highlighting the growing integration of ML and AI in the field. The USA, China, and UK were leading contributors to publications and collaborations, with institutions such as Mayo Clinic, Harvard University, and University of California playing key roles. This reflects strong global efforts in addressing heart failure challenges. However, research contributions from low- and middle-income countries remain limited, highlighting disparities that require targeted interventions (36, 37).

Highly influential journals, including Frontiers in Cardiovascular Medicine and Scientific Reports, were identified as key sources of impactful research, reflecting their role in disseminating advancements in AI and cardiology. Notable authors, such as Friedman PA, Noseworthy PA, and Attia ZI, were prominent contributors, with their works focusing on integrating AI for diagnostic precision and risk prediction. These authors were pivotal in driving the scientific discourse on leveraging computational tools to improve cardiology outcomes.

Over time, the integration of technology with cardiovascular disease research has significantly advanced. Recent literature has shown the increasing application of machine learning in cardiovascular risk prediction and diagnostic precision, including advancements in cardiac imaging technologies for early disease detection and monitoring (38–40). These advancements underscore the convergence of technology and medicine, driving innovation and reshaping cardiovascular research.

Key themes like “heart failure,” “risk,” and “artificial intelligence” illustrate the increasing reliance on ML for predictive analytics. Thematic evolution shows a shift toward advanced diagnostic technologies, such as “feature selection” and “MRI. This shift underscores the adoption of advanced technologies to enhance diagnostic precision and therapeutic strategies, aligning with modern clinical challenges as indicated by the previous studies (41–45). However, its integration into clinical practice is hindered by cost and resource constraints, particularly in low-income settings (46–48). Geographic disparities in research are a concern, with high-income countries leading AI innovation while LMICs often lack the resources to participate. Solutions include open-access datasets and North-South collaborations to improve model accuracy, build infrastructure, and ensure equitable access to AI tools (49, 50).

Similarly, while ML and AI hold promise for predictive analytics, their scalability and ethical considerations, including data privacy, algorithmic bias, and model generalizability remain key challenges (51–53). Data privacy concerns must be addressed through consent and secure data practices. Algorithmic bias can arise if models are trained on non-representative datasets, leading to biased outcomes for underrepresented populations. Ensuring diverse and inclusive datasets is essential to mitigate this risk. Furthermore, models trained on single-center data may lack generalizability, so validation across diverse settings is critical.

Emerging research areas, including proteomics and immune cell infiltration, highlight the shift toward personalized medicine in cardiomyopathy (54–56). Additionally, the focus on “telemedicine” and “cardiac imaging” in recent years reflects efforts to integrate technology into clinical workflows for remote monitoring and early diagnosis (57–59).

Citation burst analysis revealed influential studies on AI applications for cardiac arrhythmia prediction and deep learning in cardiology (60, 61). Recent bursts for cardiovascular magnetic resonance imaging (2022-2024) emphasize the growing integration of non-invasive imaging with ML algorithms to improve patient care, which aligns with the previously conducted studies (62–64).

Global collaboration networks revealed strong partnerships between leading institutions, such as Mayo Clinic, Northwestern University, and Shanghai Jiao Tong University, highlighting the interdisciplinary and international nature of this research. These collaborations are critical for addressing the multifaceted challenges of heart failure, particularly in regions with high disease burden (37, 65). However, the relatively low contributions from underrepresented regions, including Africa and parts of South Asia, underscore the need for equitable resource allocation and capacity-building initiatives (36, 66). Collaborative efforts can help standardize treatment protocols and improve access to care in low- and middle-income countries, where HF management is often inadequate (67).

Interdisciplinary teams combining computer science, cardiology, and data engineering are essential for advancing research in cardiology. By integrating clinical expertise with computational methods and data infrastructure, these teams develop machine learning models, predictive analytics, and diagnostic tools that enhance risk stratification, early detection, and personalized treatment. This collaboration has led to AI-driven solutions like ECG models and cardiac imaging, bridging technology and clinical practice to improve patient outcomes and advance precision medicine.

The prominence of AI and ML in this analysis reflects their transformative potential in addressing clinical challenges. For instance, ML algorithms for feature selection and risk prediction allow for better identification of high-risk patients, optimizing treatment strategies and improving outcomes (68–70). The alignment of these advancements with themes including precision medicine and non-invasive diagnostics demonstrates the field’s response to evolving clinical needs and technological advancements. Moreover, the recent integration of bioinformatics, genomics and proteomics into cardiology research illustrates the push toward understanding disease mechanisms at a molecular level, paving the way for novel therapeutic interventions (71–73).

In reviewing methodological trends, this analysis observed a clear shift from traditional machine learning models such as logistic regression, decision trees, and random forests toward more complex techniques including convolutional neural networks (CNNs), recurrent neural networks (RNNs), and ensemble models (61, 74, 75). This evolution, particularly notable after 2018, reflects the increasing use of high-dimensional data from ECGs, cardiac imaging, and biomarker panels in diagnosing cardiomyopathy subtypes and stratifying heart failure risk (76, 77). Diagnostic and prognostic studies in this domain frequently report area under the curve (AUC), sensitivity, specificity, and F1-score to evaluate model performance (78, 79). AUC has emerged as the most commonly used metric, especially in studies predicting ejection fraction, arrhythmia onset, and HF-related readmissions (80, 81). Recent literature has also shown a growing emphasis on explainable AI (XAI), with tools such as SHAP (Shapley Additive Explanations), LIME (Local Interpretable Model-agnostic Explanations), and attention mechanisms being adopted to improve model transparency and clinical trust. These tools are particularly valuable in cardiomyopathy and heart failure contexts, where high-stakes decision-making demands interpretability of AI outputs to guide interventions and treatment planning (82, 83)

ML and AI have shown promising clinical applications in cardiomyopathy and heart failure. ECG-based AI models for arrhythmia detection and heart failure risk stratification are among the most validated use cases, with studies demonstrating high accuracy in identifying atrial fibrillation and early signs of heart failure (60, 61). AI integration in cardiac imaging, such as MRI and echocardiography, also enhances diagnostic precision and aids in stratifying heart failure risk (84). Key barriers to clinical adoption include data standardization, as models trained on specific datasets may not generalize well to different populations. Model interpretability remains crucial for clinician acceptance, with explainable AI techniques like SHAP and LIME gaining traction to enhance transparency. Regulatory approval processes for AI models also pose significant delays, with models requiring extensive validation before receiving clinical clearance.

Despite the comprehensive nature of this analysis, certain limitations must be acknowledged. The reliance on a single database (Web of Science) may exclude relevant publications from other sources, while the focus on English-language publications could overlook contributions from non-English-speaking countries. Additionally, the temporal cutoff might underestimate the impact of recent studies, as they have had limited time to accumulate citations, which introduces a time-lag bias. Furthermore, the emphasis on quantitative metrics like citation counts may not fully capture the qualitative impact or broader clinical relevance of the research.

Future research should prioritize leveraging machine learning and artificial intelligence to enhance cardiomyopathy diagnostics, risk stratification, and treatment strategies. Key areas for development include refining ML algorithms for better predictive modeling, integrating AI with non-invasive diagnostic tools like cardiovascular magnetic resonance imaging, and developing personalized treatment protocols using ML-driven insights. Addressing challenges such as data standardization, model interpretability, and ethical concerns, particularly around patient privacy, is essential for successful implementation. Policymakers and funding agencies must support interdisciplinary collaborations that focus on AI-driven approaches, ensuring equitable access to these technologies and fostering global research efforts in cardiomyopathy.

## Conclusion

This study has systematically summarized the research status and trends in cardiomyopathy and heart failure over the last two decades using bibliometric methods. Such an analysis provides valuable insights for clinicians, researchers, and policymakers. By leveraging various analyses, the study highlights research hotspots, emerging themes, and future directions in the field. While the number of publications and collaborations continues to grow, further efforts to strengthen interdisciplinary integration via machine learning and artificial intelligence are essential to advance research and improve patient outcomes.

## Data Availability

The raw data supporting the conclusions of this article will be made available by the authors, without undue reservation.
